# The Growth Scope of Voluntary Hospitals in London

**Published:** 1897-10-09

**Authors:** 


### The Institutional Workshop.

### THE GROWTH AND SCOPE OF VOLUNTARY HOSPITALS IN LONDON.

(From a Correspondent.)

### XII.—SPECIAL HOSPITALS.

It has been shown in how curious a manner hospital construction followed popular theories and ran in grooves. First in order came the early group of general hospitals, then a whole cluster of lying-in charities for in and out patients, then came the reign of the dispensaries, and with the dawn of this century came the impulse towards specialism shown in the establishment of institutions for the cure of special diseases or cure by special methods.

The most urgent requirement of London in the direction of specialism was undoubtedly for isolation or fever hospitals. With the means then at their disposal it was entirely impracticable for hospital managers to maintain wards for infectious diseases in general hospitals without endangering the safety of all the other patients under the same roof. Yet it was impossible then as now to prevent the admission of patients suffering from other disorders who, unknown to the authorities or themselves, were in a highly infectious state. To provide a "house of recovery" for such cases and a refuge in the constantly recurring cases of patients whose houses urgently called for disinfection, separate buildings constructed for the purpose of harbouring as little as might be the deadly germs, then known by effect only, were urgently needed. It would seem that in the treatment of fever cases by isolation Dr. Rowley, of the Marylebone Workhouse and Infirmary, acted as pioneer. He and Dr. Haygarth, of Chester, experimented successfully on the poor of their respective neighbourhoods, and their methods, which lay in the direction of plentiful fresh air, whitewash, and fumigation, attracted general attention and met with speedy imitation. The first regularly established fever hospital was that known as the House of Recovery in Manchester, and in 1801 Liverpool followed the good example. It would seem that the original plan of these buildings provided accommodation for the families of infected persons until their clothing, furniture, and dwellings had been thoroughly purified, even to the "filling up the crevices where infection lodges." In 1802 a strong committee for providing a Fever Hospital in London was formed under the guidance of the wise and charitable Thomas Barnard, whose Society for Bettering the Condition of the Poor was foremost in every good movement. Delayed by the pressure of the hard times induced by the threat of invasion and many bad harvests, the project for a London House of Recovery was courageously carried through, and was, if we may judge from early statistics, more successful in the warfare against infection which it carried on in the homes of the patients than in the actual cure of the fever-stricken. Many houses at that period were settled abodes of fever, tenant after tenant being attacked, and the cases of relapse were incessant, one man and his wife living in a poor room in Drury Lane boasting of four attacks of typhus in less than a year. Many such hot-beds of fever were undoubtedly cleared away by the vigorous measures of the committee, part of the funds being appropriated for this purpose. The proportion of deaths in the hospital, if the year 1808 be taken as an average, was high, amounting to no less than 11 out of 69, or 16 per cent.; but it is mentioned that in nine of these cases the patients were almost in a hopeless condition at the time of their admission.

The year following the establishment of the Fever Hospital an attempt was made to found an institution for the special benefit of cancer patients. Dr. Denman was the moving spirit in this enterprise, and long experience among sufferers from this disease among the well-to-do had taught him the depth of despairing misery to which it reduces the poor. His design was not only charity but science. Fifty corresponding members were affiliated to the society in different parts of the world, the object being to draw into one focus every information as to symptom or remedy which could be obtained. For some reason unexplained, the society was not of long duration. A house was taken and patients received, but three years later, with its funds still unexhausted, the accounts of the institution were closed, and the balance of subscriptions was funded to await "a more favourable opportunity for renewing the progress of the charity." The work was left, in fact, to the Middlesex Hospital, where Samuel Whilbreed had in 1792 endowed a special ward for this class of patients, and where a continual extension of this noble charity took place during the early years of this century.

In 1804 the Royal Infirmary for the Eye was opened as a direct extension of the charitable work undertaken single-handed for many years by Wathen Phipps, oculist to the King, and very celebrated in his day. It appears that the immediate cause of the institution was the distress of the soldiers and sailors who took part in the Nile Expedition, and among whom a painful kind of ophthalmia, resulting, if not relieved, in total loss of sight, was very prevalent. The study of diseases of the eye had made great progress towards the end of the century, though much impeded by the dearth of any good instruments for examination. Couching for cataract was performed by several able surgeons with very good results, 84 out of 86 operations for cataract having proved successful in the year 1809. No charity has ever proved more instantaneously popular among benefactors and patients alike.

As has many times happened since, the establishment of one institution was followed immediately by a similar enterprise. In 1805 Dr. Saunders, the "shining light" of the New Finsbury Dispensary, founded the London Infirmary for the Eye. He was careful to explain that his institution was in no sense a copy of the Royal Infirmary, but had been planned out and set in hand before the promoters of that charity had conceived their enterprise. There was at that time a lamentable extent of ophthalmia among the poor in London, enfeebled as they were by poor food, and crowded together in insanitary quarters. There was ample room for both institutions, to judge from the numbers received, which in the year 1808 amounted to 2,590 patients for the Royal, and 2,747 for the London Infirmary. A very slight percentage (52 in one case, and 146 in the other) were dismissed as incurable.

Dr. Saunders' institution has not survived to the present day, but in 1816 Westminster added to its already numerous institutions the Royal Westminster Ophthalmic Hospital.

The same year, 1816, saw also the establishment of the Royal Ear Hospital and the Royal Hospital for Children and Women. Fifty years before, when the first dispensary for children was opened, it had been plausibly argued that it would be the height of cruelty to receive children as in-patients in a hospital. "If you take away a sick child from its parent or nurse you break its heart immediately, and if there must be a nurse to each child, what kind of hospital must there be to contain any number of them? Would not mothers and nurses be perpetually at variance? Would not the children disturb each other crying?" &c. Some such misgivings no doubt haunted the minds of those who planned the first children's hospital, and it was long before the modern ideal was attained under which the nurses must sometimes bear the imputation of weaning the affections of the little ones from their jealous mothers.

The establishment of the Royal Hospital for Diseases of the Chest in 1814 was another stride in the direction of specialism, and was imperatively called for by the deadly ravages of consumption in the first half of this century. Many parts of London were at that time ill-drained and marshy to a degree difficult of realisation, and insufficient food completed the work begun by the damp exhalations from the clay soil. But consumption was rife in all parts of this country, especially in the manufacturing districts, where every law of nature and humanity was openly defied for the sake of gain. The drainage of England, a gigantic work to which we owe more than can ever be estimated, was not begun till 1845, and was not completed till many years later. Even yet we are suffering under the inheritance of ague, rheumatism, and pulmonary diseases bequeathed us by our less fortunate predecessors. But, at the time of which we write, the mortality from such and kindred complaints was so appalling as to awake a just alarm with regard to the future generation, and stimulate public feeling in the direction of curative as well as preventive measures.

